# *N*-(1*H*-1,2,3-Benzotriazol-1-ylmeth­yl)phthalimide

**DOI:** 10.1107/S160053680802610X

**Published:** 2008-08-20

**Authors:** Su-Qing Wang, Fang-Fang Jian, Huan-Qiang Liu

**Affiliations:** a Microscale Science Institute, Department of Chemistry and Chemical Engineering, Weifang University, Weifang 261061, People’s Republic of China; b Department of Chemistry and Chemical Engineering, Weifang University, Weifang 261061, People’s Republic of China

## Abstract

The title compound [systematic name: 2-(1*H*-1,2,3-benzotriazol-1-ylmeth­yl)isoindole-1,3-dione], C_15_H_10_N_4_O_2_, was prepared by the reaction of 1*H*-benzotriazole and 2-bromo­methyl­isoindole-1,3-dione. The benzotriazole and isoindole units are almost planar and make a dihedral angle of 70.2 (1)° (mean planes include C and N atoms). A weak C—H⋯O intra­molecular hydrogen bond involving a carbonyl O atom as acceptor stabilizes the observed mol­ecular conformation.

## Related literature

For related literature, see: Chen & Wu (2005 ▶); Jiao *et al.* (2005 ▶).
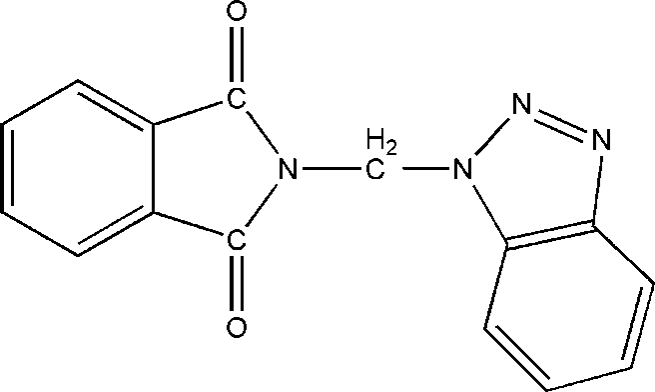


## Experimental

### 

#### Crystal data

C_15_H_10_N_4_O_2_
*M* *_r_* = 278.27
Triclinic, 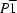

*a* = 6.9481 (11) Å
*b* = 8.0041 (13) Å
*c* = 12.030 (2) Å
α = 85.715 (3)°
β = 81.283 (3)°
γ = 73.398 (3)°
*V* = 633.38 (18) Å^3^
*Z* = 2
Mo *K*α radiation
μ = 0.10 mm^−1^
*T* = 293 (2) K
0.25 × 0.20 × 0.18 mm

#### Data collection

Bruker SMART CCD area-detector diffractometer
Absorption correction: none
3364 measured reflections
2229 independent reflections
1689 reflections with *I* > 2σ(*I*)
*R*_int_ = 0.023

#### Refinement

*R*[*F* ^2^ > 2σ(*F* ^2^)] = 0.041
*wR*(*F* ^2^) = 0.110
*S* = 1.08
2229 reflections
191 parameters
H-atom parameters constrained
Δρ_max_ = 0.16 e Å^−3^
Δρ_min_ = −0.17 e Å^−3^

### 

Data collection: *SMART* (Bruker, 1997 ▶); cell refinement: *SAINT* (Bruker, 1997 ▶); data reduction: *SAINT*; program(s) used to solve structure: *SHELXS97* (Sheldrick, 2008 ▶); program(s) used to refine structure: *SHELXL97* (Sheldrick, 2008 ▶); molecular graphics: *SHELXTL* (Sheldrick, 2008 ▶); software used to prepare material for publication: *SHELXTL*.

## Supplementary Material

Crystal structure: contains datablocks global, I. DOI: 10.1107/S160053680802610X/bh2185sup1.cif

Structure factors: contains datablocks I. DOI: 10.1107/S160053680802610X/bh2185Isup2.hkl

Additional supplementary materials:  crystallographic information; 3D view; checkCIF report

## Figures and Tables

**Table 1:** Hydrogen-bond geometry (Å, °)

*D*—H⋯*A*	*D*—H	H⋯*A*	*D*⋯*A*	*D*—H⋯*A*
C7—H7*A*⋯O1	0.97	2.55	2.890 (2)	101

**Fig. 1.**
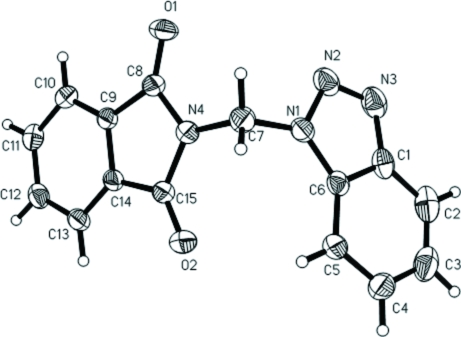
The molecular structure and atom-labeling scheme for (I), with displacement ellipsoids drawn at the 30% probability level.

## supplementary crystallographic information

### Comment

1*H*-Benzotriazole and its derivatives exhibit a broad spectrum of pharmacological activities, such as antifungal, antitumor and antineoplastic activities (Chen & Wu, 2005; Jiao *et al.*, 2005). We report here the synthesis and structure of the title compound, (I), as part of our ongoing studies on new benzotriazole compounds with higher bioactivity.

In the molecular structure of (I), bond lengths and angles are within normal ranges (Fig. 1). The dihedral angle formed by the ring 1 (N1/N2/N3/C1/C6) and the ring 3 (C1/C2/C3/C4/C5/C6) is 1.4 (1)°. The dihedral angles formed by the rings 1 and 4 (C9/C10/C11/C12/C13/C14) with the ring 2 (N4/C8/C9/C14/C15) are 69.7 (3) and 1.0 (8)°, respectively. In the phthalimide group, the C?O bond lengths are 1.201 (2) and 1.2013 (19) Å, and the C—N bond lengths are 1.400 (2) and 1.395 (2) Å. There is a C—H···O intramolecular interaction (Table 2) stabilizing the observed molecular conformation.

### Experimental

The title compound was synthesized with the following procedure: 2-bromomethyl-isoindole-1,3-dione (3.6 g, 0.015 mol) and 1*H*-benzotriazole (1.78 g, 0.015 mol) were dissolved in chloroform (15 ml). The solution was cooled to 283 K. Then, 1.5 g (0.015 mol) of triethylamine was added dropwise *via* a cannula into the well stirred solution, at 283 K. The reaction mixture was stirred at 283 K for 6 h. and at room temperature for an additional time of 16 h. Water (20 ml) was added into the solution and the resulting white solid was filtered. The organic phase was separated and dried with anhydrous potassium carbonate. The colourless organic phase was evaporated, affording (I), in 53% yield. Crystals suitable for X-ray studies were obtained from anhydrous acetone, at room temperature, after three days.

### Refinement

All H atoms were placed geometrically (C—H = 0.93 Å for aromatic CH, 0.97 Å for methylene CH_2_), and refined as riding to their parent atom with *U*_iso_(H) = 1.2*U*_eq_(C).

### Crystal data

**Table:**

C_15_H_10_N_4_O_2_	*Z* = 2
*M**_r_* = 278.27	*F*_000_ = 288
Triclinic, *P*1	*D*_x_ = 1.459 Mg m^−^^3^
Hall symbol: -P 1	Mo *K*α radiation λ = 0.71073 Å
*a* = 6.9481 (11) Å	Cell parameters from 1689 reflections
*b* = 8.0041 (13) Å	θ = 1.7–28.2º
*c* = 12.030 (2) Å	µ = 0.10 mm^−^^1^
α = 85.715 (3)º	*T* = 293 (2) K
β = 81.283 (3)º	Block, colourless
γ = 73.398 (3)º	0.25 × 0.20 × 0.18 mm
*V* = 633.38 (18) Å^3^	

### Data collection

**Table:**

Bruker SMART CCD area-detector diffractometer	1689 reflections with *I* > 2σ(*I*)
Radiation source: fine-focus sealed tube	*R*_int_ = 0.023
Monochromator: graphite	θ_max_ = 25.0º
*T* = 293(2) K	θ_min_ = 1.7º
φ and ω scans	*h* = −7→8
Absorption correction: none	*k* = −5→9
3364 measured reflections	*l* = −14→14
2229 independent reflections	

### Refinement

**Table:**

Refinement on *F*^2^	Hydrogen site location: inferred from neighbouring sites
Least-squares matrix: full	H-atom parameters constrained
*R*[*F*^2^ > 2σ(*F*^2^)] = 0.041	*w* = 1/[σ^2^(*F*_o_^2^) + (0.0531*P*)^2^ + 0.0288*P*] where *P* = (*F*_o_^2^ + 2*F*_c_^2^)/3
*wR*(*F*^2^) = 0.110	(Δ/σ)_max_ < 0.001
*S* = 1.09	Δρ_max_ = 0.16 e Å^−^^3^
2229 reflections	Δρ_min_ = −0.17 e Å^−^^3^
191 parameters	Extinction correction: SHELXL97 (Sheldrick, 2008), Fc^*^=kFc[1+0.001xFc^2^λ^3^/sin(2θ)]^-1/4^
Primary atom site location: structure-invariant direct methods	Extinction coefficient: 0.069 (7)
Secondary atom site location: difference Fourier map	

### Fractional atomic coordinates and isotropic or equivalent isotropic displacement parameters (Å^2^)

**Table:**

	*x*	*y*	*z*	*U*_iso_*/*U*_eq_	
O1	0.2735 (2)	0.98906 (15)	0.49784 (11)	0.0591 (4)	
O2	0.2515 (2)	1.39679 (17)	0.74744 (10)	0.0621 (4)	
N1	0.0501 (2)	1.05193 (18)	0.79484 (11)	0.0462 (4)	
N2	−0.0680 (3)	0.9651 (2)	0.75689 (14)	0.0626 (5)	
N3	−0.2433 (3)	1.0032 (2)	0.81950 (15)	0.0680 (5)	
N4	0.2525 (2)	1.16565 (17)	0.64463 (11)	0.0445 (4)	
C1	−0.2400 (3)	1.1155 (3)	0.89974 (16)	0.0547 (5)	
C2	−0.3877 (3)	1.1919 (3)	0.9873 (2)	0.0747 (7)	
H2B	−0.5150	1.1724	0.9984	0.090*	
C3	−0.3374 (4)	1.2959 (3)	1.0557 (2)	0.0806 (8)	
H3B	−0.4333	1.3491	1.1145	0.097*	
C4	−0.1461 (4)	1.3255 (3)	1.04057 (17)	0.0696 (6)	
H4A	−0.1176	1.3961	1.0902	0.083*	
C5	−0.0002 (3)	1.2536 (2)	0.95475 (15)	0.0528 (5)	
H5A	0.1265	1.2742	0.9437	0.063*	
C6	−0.0523 (3)	1.1476 (2)	0.88493 (14)	0.0429 (4)	
C7	0.2508 (3)	1.0372 (2)	0.73567 (15)	0.0502 (5)	
H7A	0.3062	0.9215	0.7056	0.060*	
H7B	0.3375	1.0507	0.7884	0.060*	
C8	0.2638 (2)	1.1293 (2)	0.53142 (14)	0.0431 (4)	
C9	0.2639 (2)	1.2957 (2)	0.46837 (14)	0.0402 (4)	
C10	0.2701 (3)	1.3351 (2)	0.35476 (14)	0.0486 (5)	
H10A	0.2770	1.2515	0.3035	0.058*	
C11	0.2658 (3)	1.5050 (2)	0.32021 (15)	0.0526 (5)	
H11A	0.2706	1.5361	0.2440	0.063*	
C12	0.2547 (3)	1.6289 (2)	0.39629 (16)	0.0518 (5)	
H12A	0.2528	1.7416	0.3703	0.062*	
C13	0.2461 (3)	1.5891 (2)	0.51025 (16)	0.0496 (5)	
H13A	0.2360	1.6732	0.5618	0.060*	
C14	0.2534 (2)	1.4194 (2)	0.54433 (13)	0.0407 (4)	
C15	0.2517 (2)	1.3373 (2)	0.65864 (15)	0.0436 (4)	

### Atomic displacement parameters (Å^2^)

**Table:**

	*U*^11^	*U*^22^	*U*^33^	*U*^12^	*U*^13^	*U*^23^
O1	0.0823 (9)	0.0380 (8)	0.0593 (8)	−0.0198 (7)	−0.0060 (7)	−0.0107 (6)
O2	0.0844 (10)	0.0616 (9)	0.0486 (8)	−0.0343 (7)	−0.0012 (7)	−0.0140 (7)
N1	0.0559 (9)	0.0451 (9)	0.0434 (8)	−0.0227 (7)	−0.0100 (7)	0.0027 (7)
N2	0.0816 (12)	0.0697 (11)	0.0536 (10)	−0.0451 (10)	−0.0189 (9)	0.0052 (8)
N3	0.0719 (12)	0.0840 (13)	0.0634 (11)	−0.0445 (10)	−0.0191 (9)	0.0122 (10)
N4	0.0525 (9)	0.0381 (8)	0.0426 (8)	−0.0151 (7)	−0.0009 (6)	−0.0019 (6)
C1	0.0514 (11)	0.0585 (12)	0.0545 (11)	−0.0193 (9)	−0.0101 (9)	0.0187 (10)
C2	0.0557 (13)	0.0744 (16)	0.0823 (16)	−0.0124 (12)	0.0023 (11)	0.0249 (13)
C3	0.0859 (17)	0.0606 (15)	0.0698 (15)	−0.0006 (12)	0.0246 (13)	0.0083 (12)
C4	0.1028 (18)	0.0470 (12)	0.0529 (12)	−0.0193 (11)	0.0055 (12)	−0.0028 (9)
C5	0.0687 (12)	0.0429 (11)	0.0485 (10)	−0.0211 (9)	−0.0042 (9)	0.0018 (8)
C6	0.0503 (10)	0.0377 (9)	0.0407 (9)	−0.0136 (8)	−0.0085 (8)	0.0088 (8)
C7	0.0539 (11)	0.0445 (10)	0.0498 (10)	−0.0118 (9)	−0.0045 (8)	0.0009 (8)
C8	0.0411 (10)	0.0404 (10)	0.0469 (10)	−0.0112 (8)	−0.0003 (7)	−0.0075 (8)
C9	0.0354 (9)	0.0382 (9)	0.0455 (10)	−0.0104 (7)	0.0005 (7)	−0.0040 (7)
C10	0.0478 (10)	0.0486 (11)	0.0462 (10)	−0.0108 (8)	0.0009 (8)	−0.0072 (8)
C11	0.0465 (10)	0.0565 (12)	0.0495 (11)	−0.0114 (9)	0.0007 (8)	0.0062 (9)
C12	0.0449 (10)	0.0413 (11)	0.0655 (13)	−0.0123 (8)	0.0005 (9)	0.0055 (9)
C13	0.0461 (10)	0.0405 (10)	0.0631 (12)	−0.0165 (8)	0.0015 (8)	−0.0075 (8)
C14	0.0357 (9)	0.0377 (9)	0.0483 (10)	−0.0123 (7)	0.0011 (7)	−0.0053 (8)
C15	0.0420 (10)	0.0430 (10)	0.0477 (10)	−0.0164 (8)	0.0008 (8)	−0.0088 (8)

### Geometric parameters (Å, °)

**Table:**

O1—C8	1.2013 (19)	C4—H4A	0.9300
O2—C15	1.201 (2)	C5—C6	1.390 (2)
N1—C6	1.359 (2)	C5—H5A	0.9300
N1—N2	1.361 (2)	C7—H7A	0.9700
N1—C7	1.443 (2)	C7—H7B	0.9700
N2—N3	1.299 (2)	C8—C9	1.483 (2)
N3—C1	1.375 (3)	C9—C10	1.377 (2)
N4—C15	1.395 (2)	C9—C14	1.377 (2)
N4—C8	1.400 (2)	C10—C11	1.385 (2)
N4—C7	1.446 (2)	C10—H10A	0.9300
C1—C6	1.383 (2)	C11—C12	1.377 (3)
C1—C2	1.395 (3)	C11—H11A	0.9300
C2—C3	1.360 (3)	C12—C13	1.380 (3)
C2—H2B	0.9300	C12—H12A	0.9300
C3—C4	1.398 (3)	C13—C14	1.378 (2)
C3—H3B	0.9300	C13—H13A	0.9300
C4—C5	1.365 (3)	C14—C15	1.479 (2)
			
C6—N1—N2	110.53 (15)	N4—C7—H7A	109.0
C6—N1—C7	130.37 (15)	N1—C7—H7B	109.0
N2—N1—C7	119.04 (15)	N4—C7—H7B	109.0
N3—N2—N1	108.31 (16)	H7A—C7—H7B	107.8
N2—N3—C1	108.26 (16)	O1—C8—N4	124.63 (16)
C15—N4—C8	112.24 (14)	O1—C8—C9	130.09 (16)
C15—N4—C7	124.16 (15)	N4—C8—C9	105.28 (14)
C8—N4—C7	123.55 (14)	C10—C9—C14	121.57 (16)
N3—C1—C6	109.09 (17)	C10—C9—C8	130.10 (16)
N3—C1—C2	130.8 (2)	C14—C9—C8	108.33 (14)
C6—C1—C2	120.1 (2)	C9—C10—C11	116.98 (17)
C3—C2—C1	117.1 (2)	C9—C10—H10A	121.5
C3—C2—H2B	121.4	C11—C10—H10A	121.5
C1—C2—H2B	121.4	C12—C11—C10	121.42 (17)
C2—C3—C4	122.1 (2)	C12—C11—H11A	119.3
C2—C3—H3B	118.9	C10—C11—H11A	119.3
C4—C3—H3B	118.9	C11—C12—C13	121.36 (17)
C5—C4—C3	121.7 (2)	C11—C12—H12A	119.3
C5—C4—H4A	119.2	C13—C12—H12A	119.3
C3—C4—H4A	119.2	C14—C13—C12	117.14 (17)
C4—C5—C6	116.0 (2)	C14—C13—H13A	121.4
C4—C5—H5A	122.0	C12—C13—H13A	121.4
C6—C5—H5A	122.0	C9—C14—C13	121.52 (16)
N1—C6—C1	103.80 (16)	C9—C14—C15	108.84 (15)
N1—C6—C5	133.27 (17)	C13—C14—C15	129.64 (16)
C1—C6—C5	122.91 (17)	O2—C15—N4	124.42 (16)
N1—C7—N4	112.73 (14)	O2—C15—C14	130.33 (16)
N1—C7—H7A	109.0	N4—C15—C14	105.24 (14)

### Hydrogen-bond geometry (Å, °)

**Table:**

*D*—H···*A*	*D*—H	H···*A*	*D*···*A*	*D*—H···*A*
C7—H7A···O1	0.97	2.55	2.890 (2)	101

## References

[bb1] Bruker (1997). *SMART* and *SAINT* Bruker AXS Inc., Madison, Wisconsin, USA.

[bb2] Chen, Z.-Y. & Wu, M.-J. (2005). *Org. Lett.* **7**, 475–477. 10.1021/ol047563q 15673268

[bb3] Jiao, K., Wang, Q. X., Sun, W. & Jian, F. F. (2005). *J. Inorg. Biochem.* **99**, 1369–1375. 10.1016/j.jinorgbio.2005.03.017 15869800

[bb4] Sheldrick, G. M. (2008). *Acta Cryst.* A**64**, 112–122. 10.1107/S0108767307043930 18156677

